# A Rare Case of Vocal Cord Paralysis in the Setting of Ramsay Hunt Syndrome

**DOI:** 10.7759/cureus.36027

**Published:** 2023-03-11

**Authors:** Benjamin T Gillette, Cameron M Heilbronn

**Affiliations:** 1 Otolaryngology-Head and Neck Surgery, McLaren Oakland Hospital, Pontiac, USA

**Keywords:** facial nerve paralysis, cranial neuropathies, herpes zoster oticus, vocal cord paralysis, ramsay hunt syndrome (rhs)

## Abstract

Ramsay Hunt syndrome (RHS) with concomitant vocal cord paralysis (VCP) is a rare finding. This case is particularly rare because the patient lacked the symptoms of otalgia or hearing loss when in fact, a majority of cases typically demonstrate both hearing loss and otalgia. Unique to this case is also the fact that it was complicated by a concomitant infarction of the splenium corpus callosum and a right temporal meningioma. The purpose of this study was to bring awareness to the fact that RHS can cause multiple cranial nerve neuropathies including VCP and should be included in the differential diagnosis for VCP.

## Introduction

Ramsay Hunt syndrome (RHS) is defined as herpes zoster oticus with facial nerve paralysis. This typically involves acute peripheral facial nerve paralysis with painful vesicular lesions in the concha or external auditory canal (often preceding the palsy). Typically, only 14% develop vesicles after the onset of facial weakness. Other reported symptoms of RHS include dysgeusia, hyperacusis, neurologic pain, hearing loss, and vertigo. Involvement of the vestibulocochlear nerve can lead to sensorineural hearing loss (SNHL) in 10% and vestibular symptoms in 40% of patients. Typical treatments involve antivirals/corticosteroids, analgesics, and eye protection against exposure keratitis [1]. Of note, it is important to consider malignancy in the setting of vocal fold paralysis, as this can be associated with several malignancies throughout the head, neck, and chest. These include, but are not limited to, malignancies that may involve the recurrent laryngeal nerve or vocal folds directly, such as malignancies of the skull base, neck, thyroid, larynx, and lungs [2].

## Case presentation

**Figure 1:**
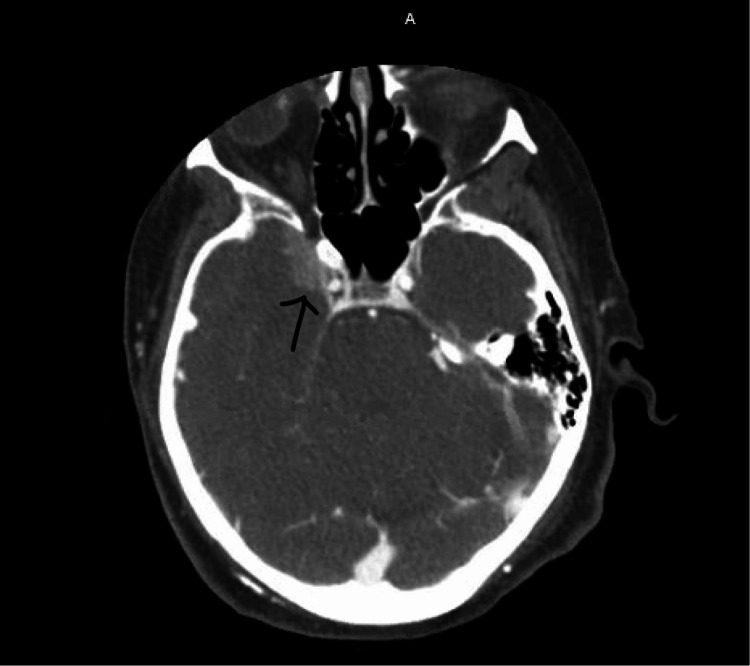
CT soft tissue head. The image demonstrates faint enhancement along medial right temporal lobe which could be reactive, infectious, low-grade glioma, meningioma, or artifact, measuring about 1.5 × 1.18 cm (arrow).

A 74-year-old female presented to the emergency department with a chief complaint of hoarseness and dysphagia after an episode of choking on a piece of chicken followed by subsequent vomiting. Gastroenterology was consulted and esophagogastroduodenoscopy (EGD) demonstrated Barrett’s esophagus, but no foreign body. After EGD, the patient was noted to have right facial droop and a code stroke was initiated followed by a computed tomography arteriogram (CTA) of the head and neck which was negative for acute bleeding but did demonstrate an area of enhancement along the medial aspect of the right temporal lobe (Figure 1).

**Figure 2:**
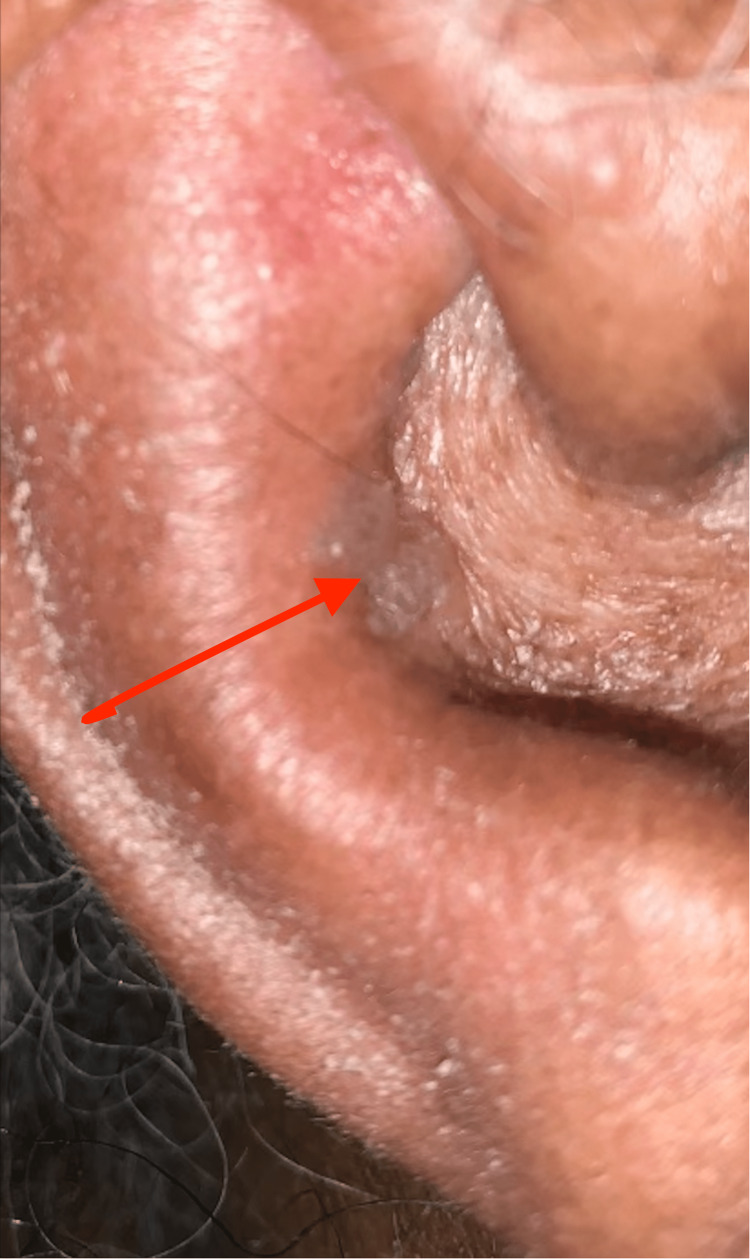
Vesicles noted along the medial aspect of the antihelix of the right ear (arrow).

Neurology then evaluated the patient and concluded this to be likely secondary to Bell’s Palsy rather than stroke. Neurology recommended magnetic resonance imaging (MRI) of the brain which also did not demonstrate signs of acute stroke. Otolaryngology (ENT) was then consulted for dysphagia. At the time of initial evaluation, the patient was found to have right-sided facial nerve paralysis (House Brackmann 6/6) of unknown duration. She had a raspy voice without signs of airway compromise. Upon further physical examination, the patient was found to have vesicles over the medial aspect of the right antihelix and a mild amount of swelling/cellulitic changes to the right auricle that was without tenderness to palpation (Figure 2).

Flexible fiberoptic laryngoscopy (FFL) demonstrated a widely patent airway with a right-sided paralyzed true vocal cord (TVC) with near complete glottic closure from compensation of the left TVC. The patient was unsure whether she had shingles in the past. The initial plan included the following: valacyclovir 500 mg three times per day for seven days, prednisone taper, artificial tears four times per day to the right eye, right eye ophthalmic lubricant/patch at night, electroneuronography (ENOG) and/or electromyography (EMG) of the facial nerve with neurology, MRI brain and neck with gadolinium if medically feasible, proton pump inhibitor twice daily, and video swallow study. Of note, MRI of the brain did demonstrate a right-sided meningioma medial to the temporal lobe as well as subacute infarction of the corpus callosum. A meningioma in this location as well as the subacute infarction of the corpus callosum are both unlikely to cause vocal cord paralysis (VCP) leading the authors to conclude the VCP was likely related to RHS causing cranial neuropathy of cranial nerve X (vagus nerve). Subsequent planning involved the patient receiving a PEG tube per gastroenterology after a failed swallow study, outpatient follow-up with ophthalmology, outpatient follow-up with otology, and plans for vocal fold medialization. The patient’s VCP persisted for four months after the initial diagnosis.

## Discussion

Ramsay Hunt syndrome (RHS) is defined as herpes zoster oticus with facial nerve paralysis. This typically involves acute peripheral facial nerve paralysis with painful vesicular lesions in the concha or external auditory canal (often preceding the palsy). Typically, only 14% develop vesicles after the onset of facial weakness. Other reported symptoms of RHS include dysgeusia, hyperacusis, neurologic pain, hearing loss, and vertigo. Involvement of the vestibulocochlear nerve can lead to sensorineural hearing loss (SNHL) in 10% and vestibular symptoms in 40% of patients. Typical treatments involve antivirals/corticosteroids, analgesics, and eye protection against exposure keratitis [1].

RHS with concomitant vocal cord paralysis (VCP) is a rare finding. A PubMed database search was performed using the search terms “Ramsey Hunt syndrome and vocal cord paralysis” with a date range of 1964-2021. The search included books and documents, clinical trials, meta-analysis, randomized controlled trials, reviews, and systematic reviews. Twenty-three total cases of RHS with concomitant VCP were discovered through this search. Of the 23 total cases discovered via literature review, only three cases explicitly stated they did not involve cranial nerve eight. These same three cases also did not explicitly state whether each patient experienced otalgia. Our case is particularly rare because the patient lacked the symptoms of otalgia or hearing loss, when in fact 20/23 cases in the literature review demonstrated both hearing loss and otalgia [2-12]. Unique to our case is also the fact that it was complicated by a concomitant infarction of the splenium corpus callosum and a meningioma. Our patient did appear to be a poor historian, at times, for example not knowing the onset of her facial nerve paralysis. Encephalopathy and associated mental status changes are reportedly associated with infarcts of the splenium of the corpus callosum [13]. These mental status changes may or may not have influenced the patient’s ability to report her otalgia or potentially skewed her audiologic findings, which may have been a limitation of this report. All cases discussed by Rasmussen et al. described RHS with multiple cranial nerve involvement. The rate of full recovery is found to be 67.7-82.9% in isolated RHS. However, when multiple cranial nerves are involved, the rate is as low as 27.3% [2]. There is extensive variability in the anatomical course of the facial nerve. This makes surgical interventions for repair of any facial nerve paralysis very difficult [14].

## Conclusions

RHS with concomitant VCP is a rare finding and should be considered in the differential diagnosis for VCP. Although the literature is lacking, multiple cases of RHS with VCP have demonstrated decreased prognosis with multiple cranial nerve involvement.

## References

[1] Pasha R, Golub JS (2014). Otolaryngology-Head and Neck Surgery: Clinical Reference Guide. Otolaryngology: Head & Neck Surgery: Clinical Reference Guide.

[2] Rasmussen ER, Mey K (2014). Vocal cord paralysis associated with Ramsay Hunt syndrome: looking back 50 years. BMJ Case Rep.

[3] Rajati M, Zarringhalam MA (2019). Ramsay Hunt syndrome associated with true vocal cord palsy- a case report. Iran J Otorhinolaryngol.

[4] Zhan J, Fu Z, Wei X (2014). Three cases of Ramsay-Hunt syndrome concurrent with ipsilateral vocal cord paralysis. [Article in Chinese]. Lin Chung Er Bi Yan Hou Tou Jing Wai Ke Za Zhi.

[5] Gómez-Torres A, Medinilla Vallejo A, Abrante Jiménez A, Esteban Ortega F (2013). Ramsay-Hunt syndrome presenting laryngeal paralysis. [Article in Spanish]. Acta Otorrinolaringol Esp.

[6] Sato K, Nakamura S, Koseki T (1991). A case of Ramsey Hunt syndrome with multiple cranial nerve paralysis and acute respiratory failure. [Article in Japanese]. Nihon Kyobu Shikkan Gakkai Zasshi.

[7] Lee HH, Yeh CW, Hung SH (2014). Ramsay Hunt syndrome with vocal fold paralysis. Kaohsiung J Med Sci.

[8] Rothschild MA, Drake W 3rd, Scherl M (1994). Cephalic zoster with laryngeal paralysis. Ear Nose Throat J.

[9] Bharadwaj S, Moffat AC, Wood B, Bharadwaj A (2016). Herpetic cranial polyneuritis mimicking brain stem infarction-an atypical presentation of Ramsay Hunt syndrome. BMJ Case Rep.

[10] Nishioka K, Fujishima K, Kobayashi H, Mizuno Y, Okuma Y (2006). An extremely unusual presentation of varicella zoster viral infection of cranial nerves mimicking Garcin syndrome. Clin Neurol Neurosurg.

[11] Benninger MS (1992). Acyclovir for the treatment of idiopathic vocal fold paralysis. Ear Nose Throat J.

[12] Maruyama R, Takenoue T, Goto K (1964). Case of herpes zoster oticus accompanied by multiple cranial nerve paralysis. [Article in Japanese]. Jibiinkoka.

[13] Sparr SA, Bieri PL (2020). Infarction of the splenium of the corpus callosum in the age of COVID-19: a snapshot in time. Stroke.

[14] Poutoglidis A, Paraskevas GK, Lazaridis N (2022). Extratemporal facial nerve branching patterns: systematic review of 1497 cases. J Laryngol Otol.

